# Association between Subretinal Drusenoid Deposits and Age-Related Macular Degeneration in Multimodal Retinal Imaging

**DOI:** 10.3390/jcm12247728

**Published:** 2023-12-16

**Authors:** Elżbieta Krytkowska, Joanna Olejnik-Wojciechowska, Aleksandra Grabowicz, Krzysztof Safranow, Anna Machalińska

**Affiliations:** 1 First Department of Ophthalmology, Pomeranian Medical University, 70-111 Szczecin, Poland; elzbietakrytkowska@gmail.com (E.K.); olejnikjoanna25@gmail.com (J.O.-W.); olagrabowicz@gmail.com (A.G.); 2 Department of Biochemistry and Medical Chemistry, Pomeranian Medical University, 70-111 Szczecin, Poland; chrissaf@mp.pl

**Keywords:** age-related macular degeneration, subretinal drusenoid deposits, choroid, pachydrusen, progression

## Abstract

Multimodal retinal imaging enables the detection of subretinal drusenoid deposits (SDD) with significantly greater accuracy compared to fundus photography. The study aimed to analyze a relationship between the presence of SDD, the clinical picture of AMD, and disease progression in a 3 year follow-up. A total of 602 eyes of 339 patients with a diagnosis of AMD, of which 121 (55%) had SDD confirmed in multimodal retinal imaging, were enrolled in the study. SDD was related to a more advanced stage of AMD (*p* = 0.008), especially with the presence of geographic atrophy (OR = 4.11, 95% CI 2.02–8.38, *p* < 0.001). Eyes with SDD presented significantly lower choroidal and retinal thickness (ATC: 210.5 μm, CRT: 277 μm, respectively) and volume (AVC: 0.17 mm^3^, CRV: 8.29 mm^3^, *p* < 0.001, respectively) compared to SDD-negative eyes (ATC: 203 μm, CRT: 277 μm; AVC: 7.08 mm^3^, 8.54 mm^3^, *p* < 0.001). Accordingly, the prevalence of pachychoroids and pachyvessels was significantly lower in the SDD present group than in eyes without SDD (*p* = 0.004; *p* = 0.04, respectively). Neither demographic factors, lipid profile, genetic predisposition, systemic vascular disease comorbidities, nor parameters of retinal vessels were affected by the presence of SDD. We found no effect of SDD presence on AMD progression (*p* = 0.12). The presence of SDD appeared to be related to local rather than systemic factors.

## 1. Introduction

Subretinal drusen deposits (SDDs) were first described over 30 years ago by Mimout et al. [1]. Since then, knowledge about these deposits has significantly expanded, but controversy remains regarding the pathomechanism as well as the risk factors associated with this type of deposit. In 2010, Zweifel et al. showed that the visual changes on ophthalmoscopy and OCT as retinal pseudodrusen are equivalent to subretinal drusenoid deposits observed in histopathological examinations [2]. The prevalence of SDD ranges from 9% to 70%, depending on the age at the stage of AMD and the use of different imaging techniques. More than 50% of these deposits may not be visualized on fundus and CFP examination, which may significantly affect the results and conclusions of the studies [3,4,5,6]. Previous studies have shown that the use of several retinal imaging techniques allows the diagnosis of SDD with significantly greater sensitivity and specificity compared to the use of CFP alone [7,8,9]. Compared to soft drusen, SDDs are presented in a different location in relation to the RPE, lying in its apical part as well as inhabiting the extrafoveal and peripheral retina, which some researchers have associated with the dysfunction of rod photoreceptors [10,11].

The results of histopathological and imaging studies indicate the vascular origin of SDD. A higher incidence of SDD was shown in patients with coronary artery disease (CAD), hypertension (HA) and higher mortality rates due to cardiovascular diseases [12,13,14,15]. Importantly, many researchers have not clarified whether their data focuses on the ocular manifestation of systemic vasculopathy leading to choroidal perfusion disorders or a symptom of a disorder originating in the choroidal microcirculation. Indeed, data show choroidal thinning in eyes with SDD, while other reports did not confirm this relationship [16,17,18,19]. Interestingly, in myopic eyes with choroidal atrophy, no increased incidence of SDD has been reported, so atrophy of this tissue does not seem to be a sufficient factor for the formation of subretinal deposits. In addition, SDD-like drusenoid deposits have also been observed in other conditions, such as Sorsby’s macular dystrophy, pseudoxanthoma elasticum, fundus albipunctatus, and vitamin A deficiency retinopathy, and are not accompanied by choroidal atrophy [20,21]. It is worth mentioning that a reduced dilatation response of the retinal arterial vessels in eyes with SDD has been documented, which suggests the role of vascular endothelial dysfunction in the pathogenesis of those lesions [22].

Considering the above information, we planned a study using multimodal retinal imaging for adequate SDD diagnosis. The objectives of the study were to assess the impact of the presence of SDD on the clinical picture of AMD and disease progression in a 3 year follow-up. Also, to assess retinal and choroidal morphology, parameters of static and dynamic analysis of retinal microcirculation in eyes with AMD, and finally, verification of the relationship between subretinal deposits and vascular diseases and specific high-risk AMD polymorphisms.

## 2. Materials and Methods

### 2.1. Subjects and Initial Management

The study included patients diagnosed with AMD in the outpatient clinic of the 1st Ophthalmological Clinic of the Pomeranian Medical University in Szczecin in 2016–2019. Demographic information, including age, sex, ocular and systemic comorbidities, drug use, smoking status, physical activity, and medical history, was obtained for each patient by a physician or trained nurse. Since an association between obesity and the presence of SDD has been previously reported, the waist/hip ratio (WHR) and body mass index (BMI) [weight (kg)/height (m)^2^] of all enrolled subjects were also assessed [15,23,24]. To assess the possible influence of smoking on the occurrence of SDD, as previously reported, the cumulative number of pack years was calculated (the average number of cigarettes smoked per day was multiplied by the number of years of smoking) [15,25,26]. Finally, each participant completed the International Physical Activity Questionnaire (IPAQ), consisting of seven questions about all types of physical activity (lasting at least 10 min) in the previous week. Physical activity scores are presented as MET min per week and calculated as previously described [27].

All participants underwent a comprehensive ophthalmological examination, including best-corrected distance visual acuity (BCDVA) assessment on the logMAR chart, Goldmann applanation tonometry (Haag-Streit Diagnostics, Koenitz, Switzerland), slit lamp biomicroscopy with detailed anterior and posterior segment evaluation, and measurement of the axial length using the IOL Master (Carl Zeiss Meditec, Jena, Germany) [28,29,30]. Multimodal retinal imaging was conducted using fundus autofluorescence (FAF), near-infrared imaging (NIR), and spectral domain (SD) OCT with enhanced depth imaging (EDI) using the Heidelberg Spectralis platform (Spectralis HRA + OCT, Heidelberg Engineering, Heidelberg, Germany). Color fundus photography was performed with a Topcon Nonmydriatic Retinal Camera (Topcon Corporation, Tokyo, Japan). Fluorescein angiography was performed in those cases where the presence of MNV could not be unequivocally confirmed by other imaging methods.

Patients with early and intermediate AMD stages in one eye underwent an ophthalmologic re-examination after 3 years to determine the presence of maculopathy progression. A total of 244 patients were initially qualified for this second stage of the study, scheduled for 2019–2020. Due to the COVID-19 pandemic and the related major disruptions in health care, 150 patients failed to attend a follow-up within 3 years or refused to participate in the project. Finally, 94 participants were re-examined, which was less than 40% of the initially recruited patients.

Patients with ophthalmic and systemic conditions that may potentially affect choroidal and retinal thickness and volume measurements, such as high refractive errors (over ± 4 diopters of spherical equivalent), glaucoma, choroiditis, retinopathy (any type), vitreomacular changes (such as an epiretinal membrane), a history of retinal detachment, or serious ocular trauma, were excluded [31,32,33,34,35,36,37,38,39]. The eyes of patients who had undergone cataract surgery within the 6 months before the study were also excluded [40].

The study was performed in accordance with the principles of the Declaration of Helsinki. All participants provided written informed consent before enrolling in the study.

### 2.2. Imaging Analysis

Enhanced depth imaging was performed on both eyes of each patient using the Heidelberg Spectralis SD-OCT (870 nm) device (Spectralis HRA + OCT, Heidelberg Engineering, Heidelberg, Germany). The A-scan rate was 70,000 scans/s, and the axial and transverse tissue resolutions were 3.9 and 6 µm, respectively. To exclude confounding factors, measurements were performed by an experienced technician after 30 min of rest at the same time of the day after mydriasis with a 1% tropicamide solution. In addition, all patients were instructed not to smoke for 6 h or drink any fluids for 1 h before the examination. During scanning, the OCT Spectralis device collected two images using a continuous double laser scan, including an infrared image from a scanning laser ophthalmoscope (SLO) and an OCT scan. The SLO images were used as references for the OCT scans. In addition, a system for actively tracking and correcting eye movements was used. To obtain SD-OCT images of the macular region, a 25° × 25° volume acquisition protocol was used to obtain 49 cross-sectional B-scans. Choroidal segmentation was performed manually after the automated retinal layer segmentation software was disabled. An experienced retina specialist moved the reference lines of the built-in automated segmentation from the retinal boundaries to the choroidal boundaries. This method allowed the use of the automatic retinal thickness and volume map features of the built-in software. The automated software version 3.1 was used to calculate choroidal volume similarly to that used for retinal volume analysis. The details of obtaining OCT images have been described previously [41].

Choroidal volume measurements were made within the 9 Early Treatment Diabetic Retinopathy Study (ETDRS) subfields, which were automatically provided by aforementioned Heidelberg Engineering software, averaged, and presented as the AV (average volume). Choroidal thickness and volume measurements from the central area according to the ETDRS map were calculated as the ATC (average thickness in the central ring area) and AVC (average volume in the central ring area). 

For a more detailed assessment of choroidal vasculature, we calculated the choroidal vascularity index (CVI). We used the semiautomated method described previously by Sonoda et al., with later modifications by Agrawal et al. [42,43]. In brief, an EDI OCT scan passing through the subfoveal region was selected and used for analysis. Images with a poorly demarcated choroidal–scleral interface (CSI) were excluded from the analysis. Binarization of the choroidal area in that scan was performed using ImageJ software (ver. 1.53e; Java 1.8.0_172 (64 bit); https://imagej.nih.gov/ij; accessed on 28 April 2021). The polygon tool was used to select the region of interest (ROI). Next, the selected region was plotted across the entire length of the EDI OCT scan to standardize the area of the ROI among all patients. The upper boundary of the ROI was manually traced along the choroid–RPE junction and the lower boundary was traced along the CSI. Application of the auto threshold was performed after conversion to eight bit images. The brightness was reduced to allow clear visualization of the choroidal vessels and minimize noise. Niblack’s autolocal threshold tool was then applied to allow demarcation of the luminal or vascular area and the stromal area. The image was then converted back to an RGB (red, green, and blue) image, and the luminal area was determined using the color threshold tool. Across all three different thresholding steps (auto threshold, Niblack’s auto local threshold, and color threshold), we used the default standard settings in the ImageJ plugin that allowed us to standardize the thresholding values for all the scans. The choroidal vascularity index was defined as the proportion of luminal area to the total choroidal area and computed for all images. Choroidal thickness, volume, and CVI measurements were all performed with a one-grader. Each measurement was performed three times and averaged. OCT scans in which it was not possible to visualize the choroidal–scleral interface were excluded from the study.

For the OCT-A scans, 512 B-scans were used to obtain a 3 × 3 mm OCT-A volume scan (10° × 10°). TruTrack Active Eye Tracking technology was enabled, which tracks the position of the eye during scanning to reduce motion artifacts or blinks. OCT-A software (Heyex Software version 1.9.201.0; Heidelberg Engineering) was used, which provided an algorithm for automatic segmentation of the retinal and choroidal layers. Manual correction of slab boundaries was performed in the case of detecting segmentation artifacts. Macular neovascularization was diagnosed when an abnormal vascular pattern was detected in the normally avascular layer on the face and/or cross-sectional OCT angiograms.

**Figure 1:**
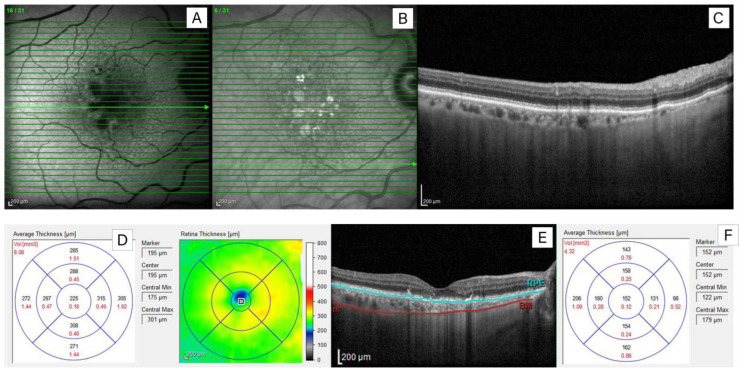
Representative images of the eye fundus of a patient with AMD with subretinal drusenoid deposits (SDD). (**A**): short-wavelength autofluorescence image; (**B**): near-infrared reflectance retinal image; (**C**): enhanced depth imaging-optical coherence tomography (EDI-OCT) scan through the perifoveal region showing drusenoid deposits in the subretinal space; (**D**): the averaged measurements of the retinal thickness (μm) (marked in black) and volume (mm^3^) (marked in red) presented for 9 ETDRS subfields and color map of the retinal thickness on the right (**E**): EDI-OCT scan of the macular region with marked retinal pigment epithelium (blue line) and scleral–uveal junction (red line); (**F**): the averaged measurements of choroidal thickness (μm) (marked in black) and volume (mm^3^) (marked in red) presented for 9 ETDRS subfields. Values of averaged volume (AV) are displayed in the upper left corner.

Fundus autofluorescence (FAF) images were obtained using an excitation blue light of 488 nm and a barrier filter beginning at 500 nm [44]. High-contrast digital near-infrared autofluorescence reflectance (NIR) images were acquired using the 815 nm diode laser of the Spectralis system. The images were captured at the time of the acquisition of the macular OCT scans. Examples of near-infrared and autofluorescence fundus images are shown in Figure 1.

The AMD classification was performed by the examining clinician according to the Ferris system [45]. Group 1 included patients with medium drusen (63–125 μm) without pigmentary abnormalities who were considered to have early AMD. Group 2 patients, who had large drusen or pigmentary abnormalities associated with at least medium drusen, were considered to have intermediate AMD. Group 3 included patients with a diagnosis of late stage AMD of both forms: advanced geographic atrophy and macular neovascularization of any type.

Soft drusen were diagnosed when yellow–white mound-like elevations with blurred boundaries and gradually reduced density from the center to the periphery were observed in CFP images, which corresponded to the moderately reflective dome-shaped sub-RPE deposits present on OCT scans [46]. Subretinal drusenoid deposits (SDD) were recognized when ≥5 discrete white–yellowish interlacing network deposits were present in the color photography, and they corresponded with a round or triangular, well-defined, hyperreflective subretinal deposit accumulation of material forming sharp peaks that may lead to abruption of the inner and outer photoreceptor segment layers on the OCT scans. On FAF and IR images, SDDs were observed as areas of hyperautofluorescence/reflectance on the center of each reticular pseudodrusen surrounded by a hyporeflective halo, forming the typical appearance of the target [47].

Pachydrusen were identified when isolated or scattered yellow–white deposits with a well-defined and complex outer border in comparison to the regular round border of soft drusen were observed in color fundus photography [48]. On the OCT images, pachydrusen were located adjacent to the pachyvessels [49]. The diagnosis of pachychoroid was established based on EDI-OCT scans in the presence of subfoveal choroidal thickness >350 µm or extrafoveal thickness exceeding the foveal choroidal thickness by at least 50 µm and in the presence of pachyvessels [50]. Pachyvessels were defined as dilated outer choroidal vessels with attenuation of the overlying choriocapillaris, and Sattler’s layers were observed on cross-section EDI-OCT images [50,51]. Only good-quality scans (Q > 30) with clearly visible choroid–scleral junctions were further analyzed.

GA was defined as a sharply demarcated hypopigmented area with visible large choroidal vessels in CFP and hypoautofluorescent vessels in FAF, with a diameter of at least 175 μm [52]. MNV was diagnosed when the presence of retinal pigment epithelial detachment (PED) associated with intra- or subretinal hemorrhages, exudatives, and/or fluid was confirmed on CFP and OCT images. The presence of pathological vessels was further confirmed with OCTA and FA (in doubtful cases). Optical tomography angiography was performed on each participant to rule out the presence of nonexudative macular neovascularization.

To assess the effect of the presence of SDD on the progression of AMD, all the abovementioned ophthalmologic examinations were repeated after 36 months. AMD progression was defined as the change in disease stage from early to intermediate AMD as well as the appearance of late stage disease features in the patient’s eyes, which were previously classified as early or intermediate in at least one eye. No AMD progression was established when none of the signs of a more advanced stage of disease were observed.

### 2.3. Retinal Vessel Analysis

The Retinal Vessel Analyzer (RVA) (Imedos GmbH, Weimar, Germany) allows for an evaluation of retinal vessel diameter by analyzing its brightness profile using video sequences obtained with a fundus camera. The exam was conducted in a dimly lit room after mydriasis with a 1% tropicamide solution, as previously described [41,53]. The examination was conducted after 15 min of rest to stabilize hemodynamic parameters. Patients were also advised to refrain from drinking coffee or smoking cigarettes for at least 5 h before the examination to exclude factors that could distort the test results. During the static vessel analysis (SVA), 30 degree retinal photographs of each subject were taken with the FF450 plus fundus camera (Zeiss AG, Jena, Germany). The images were further analyzed using VISUALIS and VesselMapsoftware (VeselMap 2, IMEDOS Systems, Ltd., Jena, Germany). The following parameters were used for the evaluation: central retinal arteriolar equivalent (CRAE), which refers to the diameter of the central retinal artery; central retinal venular equivalent (CRVE), which refers to the diameter of the central retinal vein; and arteriovenous ratio (AVR), which indicates the CRAE/CRVE ratio. The width of the selected vessels was expressed in units of measurement (UM). After the baseline vessel diameter measurement for 50 s, 3 cycles of 20 s flicker provocation for autoregulatory dilation and 80 s of steady illumination were performed, during which the vessel diameter returned to baseline. Responses from 3 cycles were averaged. Only one selected retinal artery and retinal vein were examined in each eye.

### 2.4. Serum Lipid Analysis

The total cholesterol (TC), high-density lipoprotein cholesterol (HDL), triglycerides (TG), and low-density lipoprotein cholesterol (LDL) were centrifuged (75016010, Thermo Scientific, Waltham, MA, USA) at 2000 rpm for 10 min. Next, the lipid levels were measured (L34357, Thermo Scientific, Waltham, MA, USA) by spectrophotometry.

### 2.5. Genotyping

Venous blood samples (approximately 7.5 mL) collected in EDTA tubes were centrifuged (2000 rpm, 4 °C, 10 min), and red blood cells were lysed using ammonium chloride-based lysing buffer (BD Biosciences, Franklin Lakes, NJ, USA). The nucleated cells were then counted, and DNA isolation was subsequently performed with a total DNA isolation kit (Macherey-Nagel, Düren, Germany) according to the manufacturer’s protocol. AMD risk polymorphisms were genotyped as previously described [54]. In CFH, rs1061170, encoding a Y402H interchange, was genotyped by restriction analysis with EagI, HhaI, and Hsp92II enzymes. In ARMS2, LOC387715 rs10490924, encoding an A69S interchange, was determined by direct DNA sequence analysis using an Applied Biosystems 3130 XL instrument for DNA sequencing. The genetic analysis of the PRPH2 gene was performed by exon capture with Molecular Inversion Probes (MIPs) and subsequent sequencing of amplified libraries with the Illumina NextSeq 500 system. Molecular analysis was performed by the Genomed SA bioinformatic team as well as an outwards cooperating laboratory according to generally approved standards.

### 2.6. Statistical Analysis

Qualitative variables were compared between groups with chi-squared or Fisher’s exact tests. Because the distributions of most quantitative variables were significantly different from a normal distribution, the nonparametric Mann–Whitney test was used for their analysis. A multivariate general linear regression model (GLM) adjusted for age and sex was used to determine independent factors associated with the presence of SDD in AMD patients, which were transformed logarithmically to normalize their distributions. Odds ratio (OR) and 95% confidence interval (95% CI) values were calculated for comparison of the SDD-positive group to the no SDD group, which was considered a reference. Standardized β coefficients were calculated to show the direction and strength of the associations. A *p*-value < 0.05 was considered statistically significant.

## 3. Results

### 3.1. Clinical Characteristics of the Study Group

In this study, 339 consecutive patients (602 eyes) with AMD were reviewed, of which 121 (179 eyes) were recognized to have subretinal drusen deposits, and 218 patients (423 eyes) were in the SDD absent control group. SDD-positive patients constituted 55.5% of all study participants, and subretinal deposits occurred bilaterally in 54.2% of cases. The clinical characteristics of the SDD present and absent groups are summarized in Table 1. The groups were not significantly different regarding sex or well-known atherosclerotic risk factors, including hypertension, history of ischemic heart disease, cardiac infarction, cerebral stroke, peripheral artery disease, aortic aneurysm, and smoking status. There were no significant differences in BMI, MAP, or physical activity between the groups. Similarly, total cholesterol, HDL, LDL, and triglyceride concentrations did not show significant differences between the study groups (*p* = 0.85, *p* = 0.28, *p* = 0.87, and *p* = 0.48, respectively). The patients with SDD were significantly older than those without SDD (mean: 76.95 ± 6.36 years; vs. 71.56 ± 8.17 years, *p* < 0.001, respectively).

### 3.2. Relationship between AMD Severity and SDD Presence

First, we aimed to assess the influence of SDD presence on disease characteristics. Importantly, we found a significant difference in the distribution of the AMD stage, with a higher proportion of patients with SDD in the later AMD categories (*p* = 0.008). Among participants with drusenoid deposits, 8.99% had early AMD, 46.11% had intermediate AMD, and 44.91% had late AMD, while 25.11% without SDD presented with early AMD, 33.92% had intermediate AMD, and 40.97% had late AMD. Interestingly, subretinal deposits appeared to be significantly related to soft drusen size, which accounts for the AMD grading algorithm (OR = 0.99, 95% CI 0.65–1.34, *p* < 0.001). SDD was also associated with a higher prevalence of GA in the multivariate analysis adjusted for age and sex (OR = 4.11, 95% CI 2.02–8.38, *p* < 0.001). Furthermore, central retinal thickness and volume were significantly lower in eyes with SDD than in eyes without deposits in both univariate (median CRT: 277 μm vs. 295 μm, respectively; *p* < 0.001 and median CRV: 8.285 mm^3^ vs. 8.54 mm^3^, *p* < 0.001, respectively) and multivariate analyses adjusted for age and sex (β = −0.19, *p* < 0.001 for CRT; β = −0.16, *p* = 0.0001 for CRV). Unlike GA, we did not demonstrate a relationship between the presence of subretinal deposits and the wet form of AMD (*p* = 0.51). Moreover, visual functions also did not differ significantly between the study groups (logMAR median: 0.4 for both groups, *p* = 0.21).

### 3.3. Choroidal Characteristics and SDD Presence

Next, we aimed to assess the association between RPD presence and choroidal characteristics in AMD patients (Table 2). We found that SDD presence is associated with a lower value of all measured choroidal parameters, e.g., SFCT (median: 152 μm for SDD present group and 203 μm for SDD absent group, *p* < 0.001), ATC (median: 210.5 μm for SDD present group and 294 μm for SDD absent group, *p* < 0.001), AV (median: 5.42 mm^3^ for SDD present group and 7.08 mm^3^ for SDD absent group, *p* < 0.001), AVC (median: 0.17 mm^3^ for SDD present group and 0.23 mm^3^ for SDD absent group, *p* < 0.001), and CVI (median: 0.65 for both groups; *p* = 0.05). A multivariate analysis of patients adjusted for age and sex revealed that both the SDD presence and age of the participants were variables associated with a lower AV (β = −0.19, *p* < 0.001 for SDD; β = −0.42, *p* < 0.001 for age), ATC (β = −0.22, *p* < 0.001 for SDD; β = −0.32, *p* < 0.001 for age), and AVC (β = −0.22, *p* < 0.001 for SDD; β = −0.31, *p* < 0.001 for age). Interestingly, we did not show any significant relationship between SDD and CVI in univariate (median: 0.65 for either study group, *p* = 0.051) or multivariate analysis adjusted for age and sex (β = −0.06, *p* < 0.21). Again, the only factor significantly associated with CVI was the age of the participants (β = −0.1, *p* = 0.03).

Accordingly, the prevalence of pachychoroid and pachyvessels was significantly lower in the SDD present group than in eyes without SDD (*p* = 0.004; *p* = 0.04, respectively), while pachydrusen did not coexist with SDD in either eye. In multivariate regression analyses, pachyvessels appeared to be independently related to the presence of SDD (*p* = 0.25), while the occurrence of pachychoroid features was significantly affected by both age (OR: 0.93; 95% CI: 0.89–0.97, *p* = 0.002) and subretinal deposit presence (OR: 0.21, 95% CI: 0.05–0.9, *p* = 0.04).

Representative thickness and volume maps of the retina and choroid of the eye with SDD are shown in Figure 1.

### 3.4. Relationship between SDD Presence and Retinal Vessel Characteristics

Next, we analyzed retinal vessel parameters in the study groups (Table 3). In static vessel analysis, we found both arterial and venous equivalents to be significantly lower in eyes with SDD compared to those without SDD (median: 179.3 vs. 183.4; *p* = 0.003 for CRAE; median: 209 vs. 216; *p* = 0.002 for CRVE), but AVR did not show statistically significant differences between the SDD present and absent groups (median: 0.85 for both groups; *p* = 0.93). However, multivariate regression analyses adjusted for age and sex showed no significant effect of SDD on CRAE (β = −0.04; *p* = 0.34) and CRVE (β = −0.002, *p* = 0.97). The only variable related to the lower value of retinal vessel diameter was the age of the patients (β = −0.26, *p* < 0.001 for both CRAE and CRVE). Analyzing the dynamic retinal vessel test results, we found no significant differences in arterial and venous reactivity between the study groups (DAA median: 2.9% for the SDD present group; 2.7% for the SDD absent group; *p* = 0.84; DAV median: 4.25% for the SDD present group vs. 3.9% for the SDD absent group; *p* = 0.06) (Table 3 and Table 4).

### 3.5. The Impact of SDD on AMD Progression

Next, we aimed to assess the effects of SDD presence on AMD worsening. For this purpose, 94 patients with a diagnosis of early- or intermediate-stage AMD in at least one eye were recalled over 36 months. AMD progression was defined as the change in disease stage from early to intermediate AMD as well as the presence of late stage disease features in the eyes previously classified as early or intermediate in at least one eye. During the 3 year follow-up, an increase in the severity of AMD was observed in 57.45% of patients without SDD and 42.55% of patients without these deposits, which was not a statistically significant difference (*p* = 0.12).

### 3.6. Associations between CFH Y402H and ARMS2 A69S Polymorphisms and SDD Prevalence

In the next step, we aimed to explore whether well-defined SNPs associated with an increased risk of AMD (CFH Y402H and ARMS2 A69S) are associated with SDD incidence in our patients. None of the genotypes in the two tested SNPs were associated with subretinal deposits (Table 5).

## 4. Discussion

The incidence of subretinal deposits is related to the imaging technique used to identify them. In the current study, based on multimodal imaging, the presence of SDD was confirmed in 55.5% of AMD patients, and in 52.2% of patients, the lesions were bilateral. Studies based only on color photographs of the fundus showed the presence of deposits in 6.4–18% [55,56]. The techniques with the highest sensitivity are FAF and OCT, which, when used together, show an SDD incidence rate of 52–68.8% in eyes with AMD, which is similar to the results obtained in our study [4,57]. The factor most shown to be associated with an increased incidence of SDD is the age of the patients, which is consistent with our data [58]. Importantly, we found that the gender distribution of participants did not differ between the two research groups. Indeed, some authors using multimodal retinal imaging to detect SDDs have reported a higher incidence of these deposits in women [58,59]. However, in both studies, the age of the participants in both groups was higher (median: 87 years, IQR: 81–89; mean: 83+/−3.8 years, respectively) than the age of the participants in our study. Since the mortality rate of men over 70 years of age is much higher than that of women, these results may indicate either a true association of women with SDD or an underestimation of deposit incidence in men [60,61].

It was previously shown that the incidence of SDD is related to the severity of AMD, with significant occurrences of deposits in the late stages of the disease [62,63]. In the current study, the disease stage in patients’ eyes with coexisting SDD was significantly higher than that in patients’ eyes with AMD without these deposits, and SDD appeared to be associated with the presence of geographic atrophy. The previously reported incidence of SDD in patients’ eyes with GA ranges from 36 to 90% [4,64,65]. Finger et al. showed that the presence of reticular pseudodrusen was an independent risk factor for the development of GA but not MNV [65]. Similarly, we did not observe a relationship between the presence of SDD and macular neovascularization, which was also consistent with previously published studies [66,67]. Based on the results of the longitudinal observational studies, it was shown that GA appears earlier and covers a larger area in the eyes of patients with SDD than in those without such deposits [68,69,70]. Based on the results of the HARBOR study, SDDs were proven to be a significant risk factor for macular atrophy in patients treated with ranibizumab due to MNV secondary to AMD [71]. Furthermore, the LEAD study (Laser Intervention in Early Stages of AMD) showed that the presence of SDD was a detrimental factor, leading to the acceleration of AMD progression after subthreshold nanosecond laser treatment [72]. The resolution of subretinal deposits was related to the atrophy of the external layers of the retina [73]. Significant atrophy of the retinal tissue was also clearly visible in the results of the current study. Sassmanhausen et al. demonstrated progressive outer retinal degeneration and impairment of photoreceptor function in patients’ eyes with intermediate AMD and retinal pseudodrusen over three years [74]. The presence of these deposits results in the impeded diffusion of oxygen and nutrients from the choroid into the retina and reduced removal of debris by the RPE. SDD may also exert a cytotoxic effect on the photoreceptors with which the deposits come into contact [11]. This is consistent with the results of the study, in which the presence of SDD was an independent factor associated with a decrease in retinal thickness and volume.

The results of the current study confirmed the relationship between SDD and decreases in choroidal thickness and volume in eyes with AMD [18,75,76]. This finding is in line with the data from other studies. Atrophy of the choroid was previously reported in either macular or peripapillary areas in eyes with early AMD [77,78]. Thorell et al. observed that eyes with geographic atrophy and SDD had significantly reduced choroidal thickness compared to eyes with GA and no SDD [79]. Similar observations were made in eyes with subretinal drusenoid deposits without any other features of AMD—SDD appeared to be positively associated with increasing age, female sex, and negatively associated subfoveal choroidal thickness [80]. The authors concluded that the effect of SDD on CT occurs in the early preclinical stage of AMD. In contrast, Ho et al., based on 297 eyes, showed that in multivariate analysis, including age and refractive error, the effect of SDD on choroidal parameters was not present, and they further concluded that age and refractive error, rather than RPD, were significantly associated with reduced choroidal thickness in eyes with intermediate AMD. However, the authors used single, not averaged, measurements of the choroidal thickness, contrary to our algorithm [19]. In another study, decreases in CT and CVI in all foveal, parafoveal, and perifoveal regions were shown [78]. In the current study, CVI appears not to be related to the presence of SDD in multivariate regression analysis. However, since CVI is defined as the ratio of vascular area to total choroidal area, the lack of difference in CVI between study groups with a significant reduction in choroidal thickness in SDD patients may suggest a similar reduction in choroidal intravascular area in this group. Arnold et al. hypothesized that pseudodrusen development and progression start with a diffuse loss of small choroidal vessels and diffuse choroidal thinning, followed by fibrotic replacement [81]. Studies using OCTA scans showed a decrease in choriocapillaris alteration in eyes with SDD, which is more pronounced than that observed in eyes with soft drusen [82,83,84]. Nesper et al. showed significantly larger areas of choriocapillaris nonperfusion in SDD-positive eyes than in eyes with drusen and no SDD. The authors also showed a positive correlation between the choriocapillaris (CC) nonperfusion area and visual acuity [85]. Alten et al., in an analysis of 30 eyes with SDD without other macular pathology, found that 88% of RPD cases were located within choroidal watershed zones, suggesting that choroidal hypoxia may have a role in RPD pathogenesis [86]. Since choroidal circulation is the exclusive source of blood and oxygen supply for the outer retina, its correct functioning is crucial for maintaining the functions of photoreceptor cells [87]. Therefore, any reduction in macular blood flow and changes in choroidal structure may initiate or exacerbate various pathophysiologic mechanisms. Over 7 years of follow-up, Yoon et al. demonstrated by ultrawide field imaging that the rate of RPD expansion correlates positively with the rate of subfoveal choroidal thickness reduction. Furthermore, a larger RPD area and a higher decrease in choroidal thickness were both related to the incidence of geographic atrophy. [88] Xu et al. suggested that hypoxia modulates RPE metabolism and has a role in the formation of these RPE changes [89].

An interesting finding of the current study is that subretinal drusenoid deposits did not coexist with pachydrusen in any of the eyes with AMD. Moreover, pachychoroid features and pachyvessels occurred significantly less frequently in eyes with SDD than in those without subretinal deposits. According to Spaide, the prevalence of pachydrusen is 11.7% in the Caucasian cohort, with an average subfoveal choroidal thickness of 419 µm, which is significantly higher than eyes with SDD [48]. Pachydrusen was associated with some of the entities that constitute the pachychoroid spectrum diseases [90,91]. Comparing different subtypes of retinal deposits, Nam et al. showed that choroidal atrophy as well as choroicapillaris flow voids were much more pronounced in eyes with SDD than in those with pachydrusen, which is consistent with our data [83]. Lee et al. carried out multimodal imaging of 180 eyes with polypoidal choroidal vasculopathy and found that, compared to eyes with pachydrusen, those with SDD always occurred in the context of a thin choroidal, and no choroidal vascular leakage was found in any of these eyes on indocyanine angiography images [92]. Moreover, studies have shown that pachydrusen, similar to other retinal deposits, undergoes dynamic changes, but unlike SDD and soft drusen, RPE and retinal atrophy are not observed after pachydrusen resorption [93]. Considering the above, it can be suspected that SDD is the result of different pathological processes than pachydrusen.

The results of the current study did not show any significant differences in the frequency of co-occurrence of certain systemic vascular diseases and their risk factors between the two study groups. Accordingly, we found no dissimilarities in serum cholesterol and lipoprotein levels, smoking status, obesity prevalence, or physical activity levels between the groups with and without SDD. This indicates that these specific systemic disorders are unrelated to the pathogenesis of SDD in AMD eyes. These observations are in line with previous reports. In the population-based Montrachet study conducted by Gabrielle et al. based on the analysis of multimodal images of 1500 participants, SDD was not associated with systemic vascular disorders [80]. Furthermore, McCarter et al., in a study of 534 participants using ultrawide imaging of the retina, did not confirm any association between coronary artery disease and RPD [94]. Wu et al. analyzed patients with a diagnosis of intermediate AMD and found no significant difference in sex distribution, smoking history, or cardiovascular factors, including hypertension, atherosclerosis, and hypercholesterolemia, between eyes with and without SDD [26]. On the other hand, reports show an association between SDD and high-risk vascular disease [15,59,95,96,97]. This discrepancy may be due to several reasons. First, the definition of high-risk vascular diseases differed between the studies. In addition, some authors analyzed only eyes with intermediate AMD, and diabetic patients were included in their study, which may also confuse the results [95]. Some authors included study participants with and without the features of AMD [13]. In addition, the mean age of the participants was significantly lower than ours [13]. Furthermore, SDD presence was determined in some reports using only stereoscopic color fundus photographs [15].

In the current study, there was no link between SDD and retinal vessel diameters or reactivity. These parameters indirectly indicate the function of the vascular endothelium, and their disturbance in arterial hypertension, atherosclerosis, and CAD has been previously demonstrated [98,99,100,101]. Interestingly, Rabiolo et al. observed a reduced dilation response of the retinal arteries in eyes with SDD [22]. However, the study included only a small group of 23 participants with SDD and without AMD; thus, the results cannot be cross-referenced with our data.

The results of the current study did not show any differences in the distribution of high-risk AMD variants in the ARMS2 and CFH genes between the SDD-positive and SDD-negative groups. However, research results in this area are ambiguous. Meta-analyses are showing a stronger contribution of ARMS2 in the AMD with SDD groups versus the AMD without SDD groups in comparison with CFH genotypes [102,103]. Similarly, Finger et al., based on the results of a study of over 20,000 participants, showed that high-risk polymorphisms in either CFH or ARMS were related to retinal pseudodrusen presence in the eyes of patients with AMD [67]. Accordingly, the results of the Blue Mountains Eye Study showed that the presence of risk alleles of CFH-rs1061170 or ARMS2-rs10490924 were both associated with higher reticular drusen incidence in the 15 year follow-up [104]. In contrast, Smith et al. showed that the CFH Y402H polymorphism is related to a decreased risk of RPD, while ARMS2 A69S is associated with an increased risk of the presence of these deposits [105]. The possible reason for these discrepancies is that in all the abovementioned studies, the diagnosis of subretinal deposits was made exclusively by analyzing color fundus photography, contrary to our multimodal algorithm. Wu et al., using multimodal imaging, reported a higher frequency of ARMS2 variants in patients’ eyes with retinal pseudodrusen than in those without deposits in patients with intermediate AMD [26]. Similarly, Ueda-Arakawa et al. also found the ARMS2 A69S risk allele to be related to the occurrence of RMD in a Japanese population. However, the results could not be simply extrapolated for Europeans [9]. The results of the ALIENOR study based on 472 French patients showed that the presence of the allelic variants rs10490924 for ARMS2 and rs1061170 for CFH was associated with a higher incidence of SDD in the 5 year follow-up [106]. However, Puche et al. found none of the AMD risk alleles to be associated with the development of reticular macular disease (RMD) diagnosed on infrared and SD-OCT imaging [107]. Spaide et al. compared the eyes of patients with SDD and AMD and showed a similar frequency of CFH Y402H and ARMS2 A69S alleles in the studied groups [108].

## 5. Conclusions

In conclusion, in the present study, SDD was not associated with systemic vascular disease or its risk factors. The lack of impact of SDD on the function of the vascular endothelium assessed in the morphological and functional tests of the retinal vessels confirms the aforementioned information. This suggests that the presence of subretinal deposits may be influenced by local rather than systemic factors. Accordingly, the presence of SDD appeared to be unrelated to genetic background. Drusenoid deposits are much more common in eyes with an advanced stage of the disease and geographic atrophy. This may indicate a drusen-independent mechanism associated with defects within the RPE, Bruch’s membrane, or choroid. Indeed, in patients’ eyes with AMD, SDDs are associated with retinal and choroidal atrophy. Despite the abundance of clinical studies, the phenomenon of subretinal drusenoid deposits has not been fully explained, and a clear evaluation of the results of our studies in the context of other studies is difficult due to the use of different methods of their detection. Therefore, long-term population studies using multimodal imaging are needed to capture those factors that are associated with the development of SDD.

## Figures and Tables

**Table 1:** Characteristics of the patients in the study groups. Statistical significance was established at *p* < 0.05 (bolded).

Parameter	SDD Present	SDD Absent	*p*-Value *
Number of subjects	121	218	-
Sex (male/female) [%]	40/60	33.6/66.4	0.28
Age [years] (mean ± SD)	76.95 (6.36)	71.56 (8.17)	**<0.001**
Hypertension [%]	70.75	61.58	0.13
Duration of hypertension [years] (mean ± SD)	9.62 (10.86)	7.76 (8.94)	0.16
History of ischemic heart disease [%]	16.04	17.46	0.87
Duration of ischemic heart disease [years] (mean ± SD)	1.36 (4.64)	1.29 (4.14)	0.41
History of myocardial infarction [%]	6.6	6.88	1.0
Stroke [%]	3.81	1.59	0.25
Peripheral artery disease [%]	6.6	4.76	0.59
Aortic aneurysm [%]	1.9	1.6	1.0
Current smokers [%]	9.43	16.32	0.12
Former smokers [%]	50.1	51.58	0.81
Period without smoking [years] (mean ± SD)	7.75 (11.65)	5.9 (9.98)	0.21
Smoking pack years (mean ± SD)	14.93 (19.87)	13.37 (19.2)	0.73
BMI [kg/m^2^] (mean ± SD)	26.98 (4.44)	26.88 (4.23)	0.52
WHR [arbitrary units] (mean ± SD)	0.9 (0.1)	0.9 (0.09)	0.71
MAP [mmHg] (mean ± SD)	98.22 (11.05)	97.9 (11.18)	0.85
Physical activity [MET] (mean ± SD)	1200.35 (1155.42)	1723.77 (2444.57)	0.12
Total cholesterol [mg/dL] (mean ± SD)	202.4 (43.55)	204.21(44.62)	0.85
HDL [mg/dL] (mean ± SD)	58.6 (12.69)	61.13 (14.97)	0.28
LDL [mg/dL] (mean ± SD)	119.5 (38.93)	118.97 (38.82)	0.87
TG [mg/dL] (mean ± SD)	101.32 (43.31)	108.04 (55.31)	0.48

WHR—waist-hip ratio; MAP—mean arterial pressure; HDL—high-density lipoprotein; LDL—low-density lipoprotein, TG-triglycerides. * Mann–Whitney U test/Fisher’s exact test.

**Table 2:** Clinical factor differences between eyes with and without SDD. Statistical significance was established at *p* < 0.05 (bolded).

Clinical Feature		SDD Present	SDD Absent	*p*-Value *
AMD stage	Early (yes [%])	8.99	25.11	**0.008**
	Intermediate (yes [%])	46.11	33.92	
	Late (yes [%])	44.91	40.97	
Geographic atrophy (Yes/No)		24/53	18/181	**0.00003**
MNV (Yes/No		39/16	112/60	0.51
Pachychoroid (Yes/No)		2/175	34/430	**0.004**
Pachyvessels (Yes/No)		33/145	125/339	**0.035**
Pachydrusen (Yes/No)		0/178	38/428	**0.0002**

MNV—macular neovascularization; * Mann—Whitney/Fisher’s exact test.

**Table 3:** Differences in choroidal and retinal parameters between groups with and without subretinal drusenoid deposits (SDDs). Statistical significance was established at *p* < 0.05 (bolded).

Clinical Parameter	SDD Present Median (IQR)	SDD Absent Median (IQR)	*p*-Value *
Visual acuity (logMAR)	0.4 (0.5)	0.4 (0.52)	0.21
SFCT (μm)	152 (113)	203 (138)	**<0.001**
ATC (μm)	210.5 (144)	294 (136)	**<0.001**
AV (mm^3^)	5.42 (2.57)	7.08 (3.29)	**<0.001**
AVC (mm^3^)	0.17 (0.12)	0.23 (0.11)	**<0.001**
CVI	0.65 (0.04)	0.65 (0.05)	**0.05**
CRT (μm)	277 (64)	295 (104)	**<0.001**
CRV (mm^3^)	8.29 (0.78)	8.54 (0.88)	**<0.001**
CRAE	179.3 (20.3)	183.4 (22.1)	**0.003**
CRVE	209 (26.65)	216.5 (27.8)	**0.002**
AVR	0.85 (0.11)	0.85 (0.09)	0.93
DAA (%)	2.9 (3.2)	2.7 (3.1)	0.84
DAV (%)	4.25 (2.5)	3.9 (2.7)	0.06

SFCT—subfoveal choroidal thickness, ATC—averaged thickness center of the choroid; AV—averaged volume of the choroid; AVC- averaged volume center of the choroid; CVI—choroidal vascular thickness, CRT—central retinal thickness, CRV = central retinal volume; CVI—choroidal vascular index; CRT—central retinal thickness; CRV—central retinal volume; CRAE—central retinal artery equivalent; CRVE—central vein artery equivalent; AVR—arteriovenous ratio, DAA—dynamic analysis of arteries, DAV—dynamic analysis of veins. The values are presented as the median (IQR). * Mann—Whitney/Fisher’s exact test.

**Table 4:** Differences in choroidal and retinal parameters between groups with and without subretinal drusenoid deposits in multivariate logistic analysis adjusted for age and sex. Statistical significance was established at *p* < 0.05 (bold).

Clinical Parameter	SFCT	ATC	AV	AVC	CVI	CRT	CRV	CRAE	CRVE
β	−0.14	−0.22	−0.19	−0.22	−0.06	−0.19	−0.16	−0.04	−0.002
*p*-value	**0.002**	**<0.001**	**<0.001**	**<0.001**	0.21	**<0.001**	**<0.001**	0.34	0.97

SFCT—subfoveal choroidal thickness, ATC—average thickness center, AV—average volume, AVC—average volume center, CVI—choroidal vascular thickness, CRT—central retinal thickness, CRV—central retinal volume, CRAE—central retinal artery equivalent, CRVE—central retinal vein equivalent.

**Table 5:** Distribution of different high-risk AMD genotypes according to the presence of subretinal drusenoid deposits (SDDs).

Tested SNP	Genotype	% of AMD Patients with SDD	% of AMD Patients without SDD	*p*-Value *
*CFH* Y402H	TT	35.71%	64.29%	0.92
	TC	35.43%	64.57%	
	CC	38.04%	61.96%	
*ARMS2* A69S	GG	37.50%	62.50%	0.89
	GT	34.93%	65.07%	
	TT	38.10%	61.90%	

* Chi-squared test.

## Data Availability

The data that support the findings of this study are available upon reasonable request from the corresponding author, A.M.
